# Turning an old *GADD*get into a troublemaker

**DOI:** 10.1038/s41418-018-0087-6

**Published:** 2018-03-06

**Authors:** Daria Capece, Daniel D’Andrea, Daniela Verzella, Laura Tornatore, Federica Begalli, Jason Bennett, Francesca Zazzeroni, Guido Franzoso

**Affiliations:** 1 Centre for Cell Signalling and Inflammation, Department of Medicine, Imperial College London, London, W12 0NN UK; 2 Department of Biotechnological and Applied Clinical Sciences, University of L’Aquila, L’Aquila, 67100 Italy

Life must continually defend against infection. One of the most ancient types of immunity is the process of active cell death, which first arose in evolution plausibly with protists, as a primordial defence against intracellular parasites. Owing to this common ancestral origin, there is remarkable symmetry and overlap in proteins, signalling complexes and domains between the pathways of apoptosis and those of inflammation. The clearest paradigms of this interconnection and overlap are caspase-family proteases, which can signal to cell death or the activation of proinflammatory cytokines, and NF-κB-family transcription factors, the ubiquitous rapid-response system centrally involved in the regulation of apoptosis and immune and inflammatory responses, in both vertebrates and invertebrates [1].

Given their central role in disease pathogenesis, the mechanisms underpinning these pathways are of paramount clinical significance. Notably, in many cancer types, the normally tight regulation of apoptosis and inflammation is frequently disrupted by an aberrant NF-κB activation [2]. While multiple control mechanisms normally ensure the prompt cessation of physiological NF-κB signalling, in most human cancers, numerous alterations constitutively activate NF-κB, enabling it to fuel tumour progression, metastatic dissemination and therapy resistance by regulating genes that suppress cancer-cell apoptosis and orchestrate inflammation in the tumour microenvironment (TME), thus hijacking the ancient connection between apoptotic and inflammatory pathways [2, 3].

Despite intense investigation, the mechanisms by which NF-κB mediates these functions in oncogenesis remain incompletely understood. Recently, we furthered their understanding in multiple myeloma, a cancer where plasma cells become addicted to NF-κB activation for survival. We showed that oncogenic NF-κB signalling mediates this effect by upregulating its pro-survival target gene, *GADD45B*, a member of the GADD45-gene family. Consequently, GADD45β is highly expressed in most multiple myelomas, where it suppresses apoptosis ensuing from spontaneous JNK/MAPK-pathway activation by inhibiting the JNK kinase, MKK7 [4].

In addition to blocking cancer-cell apoptosis, oncogenic NF-κB signalling operates in the TME, thereby linking cancer to inflammation [3]. NF-κB activation in non-malignant tumour-associated cells, especially those of the myeloid lineage, has been shown to enhance the production of cytokines and other specialised effectors that promote tumour-cell proliferation, tissue invasion and therapy resistance, while suppressing anti-tumour immune responses [5]. However, the notion that NF-κB would mediate its oncogenic functions in malignant cells and the TME via entirely different gene sets, which either suppress apoptosis or govern inflammation, is at odds with the overall architectural conservation and co-evolution of these processes.

Recently, we demonstrated that this is indeed not the case [6]. We identified a dedicated axis of the NF-κB pathway, mediated by GADD45β, which integrates the NF-κB-dependent mechanism suppressing tumour-cell apoptosis with that governing tumour-based inflammation [4, 6]. We showed that macrophage-associated GADD45β expression governs an innate-immunity checkpoint restricting TME-based inflammation and T-lymphocyte trafficking into tumours, the major barrier to effective immunotherapy [6, 7]. Correspondingly, in animal models of multiple solid cancers, myeloid-restricted *Gadd45b* ablation restored pro-inflammatory tumour-associated macrophage (TAM) activation and intratumoural immune-cell infiltration, leading to TME-based tertiary lymphoid-structure (TLS) formation, reactivation of anti-tumour immune responses and diminished oncogenesis [6]. Interestingly, GADD45β mediates these diverse oncogenic functions through distinct mechanisms, which suppress cancer-cell apoptosis by inhibiting MKK7/JNK activation and curb inflammation by attenuating macrophage-associated p38 signalling [4, 6].

From an evolutional perspective, it would seem advantageous to unify anti-apoptotic and anti-inflammatory pathways into an integrated mechanism relying on new gene transcription, e.g. the GADD45β-mediated mechanism, as a defence against intracellular pathogens such as viruses. Such a mechanism would monitor the transcriptional fitness of the cell, as a cue that the cell is healthy or has cleared the infection, and make it a prerequisite for cell survival and, concurrently, terminating inflammation. Conversely, if a virus has taken over the cellular biosynthetic machinery to serve its reproductive needs, the same mechanism would detect the infection and doom the cell to die, while enabling the immune reaction to continue.

In keeping with this evolutional theme, GADD45 proteins share homology with the L7Ae/L30e/S12e/GADD45 RNA-binding domain found in ribosomal and ribonucleoprotein particle (RNP)-associated proteins in eukarya and archaea [8, 9]. An ancient connection between ribosomes and defence mechanisms is also suggested by the involvement of ribosomal proteins in cell-death signalling and immunity, and that of ribosomal protein S3 as a core subunit of NF-κB complexes [10, 11]. This connection likely stems from the integral involvement of ribosomes in both viral replication and antiviral responses mediated by translation arrest and RNP aggregation into stress granules [12]. This raises new questions concerning the evolution and biology of GADD45 proteins. For example, did they arise as ribosomal components that later evolved into RNP-associated defence mechanisms? Did their cap-independent translation and role in DNA demethylation subsequently arise as immune mechanisms capable of operating during an infection [12, 13]?

Irrespective of when and how the different GADD45β functionalities first arose in evolution or were controlled by NF-κB, the integration of the mechanisms governing apoptosis and inflammation by the NF-κB/GADD45β axis has far-reaching therapeutic implications. Indeed, despite the long-lasting effort, developing a clinically useful NF-κB inhibitor has not proven possible, due to the dose-limiting toxicities of globally suppressing NF-κB [3, 14, 15].

We sought to overcome this problem in multiple myeloma by alternatively targeting the GADD45β/MKK7 signalling module in a non-redundant, cancer-restricted survival axis of the NF-κB pathway. We demonstrated that GADD45β/MKK7-targeting agents are effective in killing multiple myeloma cells, *ex vivo* and *in vivo*, and, crucially, are not toxic to healthy tissues, as they preserve the physiological functions of NF-κB [4]. Due to this cancer-selective mode of action, GADD45β/MKK7 inhibitors demonstrated no adverse effects, alongside a cancer-selective pharmacodynamic response, in their first-in-human study in refractory multiple myeloma patients. Notably, our finding that GADD45β also mediates a TME-based innate-immunity checkpoint provides an attractive therapeutic route downstream of NF-κB to reactivate anti-tumour immune responses.

Indeed, while immunotherapies are revolutionising the clinical management of certain malignancies, the majority of cancer patients fails to respond to these therapies, due to additional TME-mediated mechanisms that exclude T cells from tumours or cause T-cell exhaustion [7]. An attractive approach to overcome this barrier would be to reactivate TME-based innate immunity. While no NF-κB inhibitor is clinically approved for this purpose, the encouraging results from the first-in-human study of GADD45β/MKK7 inhibitors suggest that the myeloid-associated GADD45β-dependent checkpoint will be similarly amenable to therapeutic intervention for re-programming TAMs to unleash TME-based inflammation and redirect CD8^+^ T-cell trafficking into tumours. Therefore, innate immunotherapies targeting GADD45β could plausibly overcome immunotherapy resistance, increasing response rates in otherwise refractory cancer patients.

**Fig. 1:**
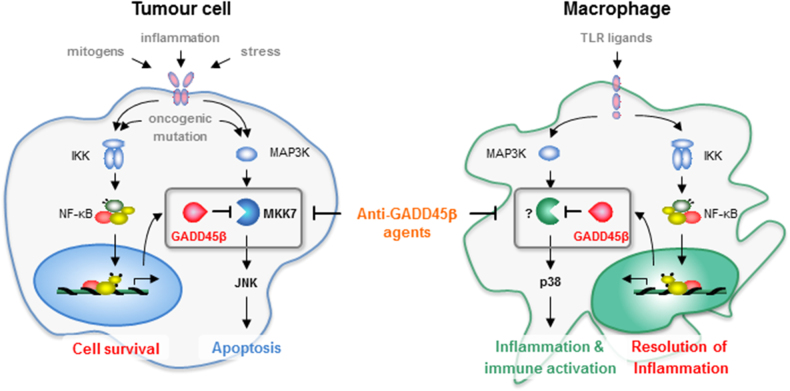
Schematic representation of the dual functions of GADD45β in oncogenesis. GADD45β mediates its distinct oncogenic functions through separate tissue-specific mechanisms, which promote cancer cell survival by inhibiting sustained MKK7/JNK activation (left) and suppress TME-based inflammation, pro-inflammatory TAM activation and CD8^+^ T-cell recruitment into tumours by attenuating p38 signalling in myeloid cells (right). Also shown is how GADD45β-targeting therapeutic agents could provide an effective means of countering oncogenesis by promoting TME-based inflammation and adaptive anti-tumour immune responses (right) and, at the same time, inducing malignant cell apoptosis (left), thus affording a dual clinical benefit.

Further studies will determine the clinical benefit of combining conventional and GADD45β-targeting immunotherapies. This notwithstanding, our findings suggest that targeting the NF-κB pathway through GADD45β would provide an effective means to counter oncogenesis by reversing TME-mediated immunosuppression and, concurrently, inducing cancer-cell apoptosis, thereby providing dual therapeutic benefit (Fig. 1). The widespread correlation between *GADD45B* expression and poor clinical outcome in human cancers underscore the general clinical significance of the NF-κB-dependent mechanisms mediated by GADD45β in oncogenesis [4, 6]. Only time will tell whether the therapeutic opportunity presented by the GADD45β-mediated signalling axis will turn out to be gold or just flashes in the pan. What is certain, though, is that disentangling the complex mechanisms and networks underpinning the role of NF-κB in oncogenesis will move the field closer to seizing the treasure trove of treatments still hidden in the NF-κB pathway and ultimately solve its biology.
